# Long-term outcomes of semi-implantable functional electrical stimulation for central drop foot

**DOI:** 10.1186/s12984-019-0542-8

**Published:** 2019-06-11

**Authors:** Lars Buentjen, Andreas Kupsch, Imke Galazky, Roman Frantsev, Hans-Jochen Heinze, Jürgen Voges, Janet Hausmann, Catherine M. Sweeney-Reed

**Affiliations:** 1 Dept. of Stereotactic Neurosurgery, Otto-von-Guericke University Magdeburg, University Hospital Magdeburg, Leipziger Str. 44, 39120 Magdeburg, Germany; 2 Dept. of Neurology, Otto-von-Guericke University Magdeburg, Leipziger Str. 44, 39120 Magdeburg, Germany; 3 Neurology Moves, Bismarckstr. 45, 10627 Berlin, Germany; 4 Leibniz Institute for Neurobiology, Magdeburg, Leipziger Str. 44, 39120 Magdeburg, Germany; 5 Neurocybernetics and Rehabilitation, Dept. of Neurology, Otto-von-Guericke University Magdeburg, University Hospital Magdeburg, Leipziger Str. 44, 39120 Magdeburg, Germany; 6 German Center for Neurodegenerative Diseases, 39120 Magdeburg, Germany

**Keywords:** Foot drop, Gait, Peroneal nerve stimulation, Functional electrical stimulation, Semi-implantable, Actigait

## Abstract

**Background:**

Central drop foot is a common problem in patients with stroke or multiple sclerosis (MS). For decades, it has been treated with orthotic devices, keeping the ankle in a fixed position. It has been shown recently that semi-implantable functional electrical stimulation (siFES) of the peroneal nerve can lead to a greater gait velocity increase than orthotic devices immediately after being switched on. Little is known, however, about long-term outcomes over 12 months, and the relationship between quality of life (QoL) and gait speed using siFES has never been reported applying a validated tool. We provide here a report of short (3 months) and long-term (12 months) outcomes for gait speed and QoL.

**Methods:**

Forty-five consecutive patients (91% chronic stroke, 9% MS) with central drop foot received siFES (Actigait®). A 10 m walking test was carried out on day 1 of stimulation (T1), in stimulation ON and OFF conditions, and repeated after 3 (T2) and 12 (T3) months. A 36-item Short Form questionnaire was applied at all three time points.

**Results:**

We found a main effect of *stimulation* on both maximum (*p* < 0.001) and comfortable gait velocity (p < 0.001) and a main effect of *time* (*p* = 0.015) only on maximum gait velocity. There were no significant interactions. Mean maximum gait velocity across the three assessment time points was 0.13 m/s greater with stimulation ON than OFF, and mean comfortable gait velocity was 0.083 m/s faster with stimulation ON than OFF. The increase in maximum gait velocity over time was 0.096 m/s, with post hoc testing revealing a significant increase from T1 to T2 (*p* = 0.012**)**, which was maintained but not significantly further increased at T3. QoL scores showed a main effect of *time* (*p* < 0.001), with post hoc testing revealing an increase from T1 to T2 (p < 0.001), which was maintained at T3 (p < 0.001). Finally, overall absolute QoL scores correlated with the absolute maximum and comfortable gait speeds at T2 and T3, and the increase in overall QoL scores correlated with the increase in comfortable gait velocity from T1 to T3. Pain was reduced at T2 (p < 0.001) and was independent of gait speed but correlated with overall QoL (p < 0.001).

**Conclusions:**

Peroneal siFES increased maximal and comfortable gait velocity and QoL, with the greatest increase in both over the first three months, which was maintained at one year, suggesting that 3 months is an adequate follow-up time. Pain after 3 months correlated with QoL and was independent of gait velocity, suggesting pain as an independent outcome measure in siFES for drop foot.

## Introduction

Drop foot is a common symptom in patients suffering from first motor neuron lesions, such as due to stroke and multiple sclerosis (MS). It is characterized by impaired lifting of the forefoot from the ground during the swing phase of walking and by a lack of stability during the early stance phase. Drop foot results in an altered gait pattern [3] and increased risk of falls [8]. Application of an ankle foot orthosis (AFO) is the traditional approach to improving gait pattern and reducing falls. However, it is not well-tolerated in all patients [10]. In recent years, gait improvement has been achieved using functional electrical stimulation (FES) [1, 10, 16, 23, 25], which combines the orthotic benefits of an AFO with a more physiological approach that involves muscle contraction and the related sensory feedback [10, 25]. Transcutaneous FES (tcFES) of the peroneal nerve has been associated with significantly reduced falls compared to intensive physiotherapy [7]. Indeed, 69% of the falls in this FES group occurred when the system was not used. Moreover, a systematic review of FES in MS patients indicates increased gait speed using FES [19]. Semi-implantable FES (siFES) of the peroneal nerve has been found to increase gait speed and improve gait patterns compared with a baseline without stimulation [6, 10, 17], compared to orthotic devices [1, 23], and also compared to tcFES [17]. The findings of a systematic review, including predominantly chronic stroke patients, however, did not suggest a difference between tcFES and siFES in terms of walking speed [13]. An implantable stimulator does, however, offer the advantage of avoiding the need for daily optimization of stimulator location [28] and potential skin lesions associated with surface stimulation electrodes. Moreover, the possibility of using a 4-channel implantable system, with independent control of each channel, means that the volume of tissue activated within the nerve can be individually selected, in order to optimize dorsiflexion of the foot while avoiding stimulation of the sensory fascicles of the common peroneal nerve [10]. Here we retrospectively hypothesised that increases in gait speed are associated with improvements in quality of life (QoL). Furthermore, we assumed pain scores had improved under therapy and expected them to be related to the overall QoL, and we hypothesised that increased gait velocity would have resulted in improvement of both physical and emotional subscores of the QoL. To address these hypotheses, we evaluated improvement in gait velocity in the largest cohort of patients to date, with stimulation ON and OFF, at three time points over 1 year, to assess the short- and long-term effects of siFES, examining correlation between gait speed and QoL, as well as between changes in these factors, over a year of continuous treatment.

Most studies of implantable systems for stroke to date cover observation periods of 3 to 6 months post-surgery and suggest siFES provides a promising approach to managing drop foot. An increase in gait velocity and endurance, as well as an improvement in QoL, was observed 3–6 weeks post-operatively in a cohort of 27 patients receiving siFES [17]. Trials applying tcFES, which has been available since the early seventies [27], have tended to employ standardized and stratified re-examination, with early and long-term follow-up periods, such as 6 and 12 weeks [16], 3 and 12 months [25], and 24 days and 3 years [28]. A recent long-term multi-centre study applying siFES reported an improved gait pattern in a cohort of 10 stroke patients 6 months following siFES activation and in a separate cohort of 12 stroke patients 1 year after activation [1]. Their findings suggested greater knee stability, ankle plantarflexion power, and propulsion than that provided by an AFO. Here, we examined both the short- and long-term effects of using multichannel peroneal siFES in the largest patient group thus far reported, including both stroke and MS patients. The independent association between slow gait velocity and an increased risk of falls [8] renders gait velocity a valid surrogate parameter for the orthotic functionality of devices aiming to improve the limitations of drop foot. We aimed to investigate whether gait velocity improvements translate into QoL changes. Long-term follow-up (one year or longer) has been reported for large cohorts (more than 20 patients) using tcFES [25, 28], and for a smaller cohort (*N* = 12) using siFES [1]. Long-term follow-up in a large cohort of patients receiving siFES and evaluating QoL has not yet been reported. The particular strengths of the current study are the large cohort, the inclusion of short- and long-term follow-up, and the evaluation of QoL and its correlation with gait speed.

## Methods

### Patient characteristics and selection

Forty-five consecutive patients with central drop foot, who benefited from surface peroneal FES, received a semi-implantable system (Actigait®, Otto Bock, Duderstadt, Germany) [2]) consisting of an implantable 4-channel peroneal cuff electrode-antenna assembly controlled by an external pulse generator, driven by an external heel switch (illustrated in [10]).

All patients suffered from “drop foot” due to a first motor neuron lesion. The study is a retrospective analysis of clinical data, and the patients had consented to analysis and publication of their anonymized data. The study assessments were a part of routine clinical assessment. Clinical characteristics are provided in Table 1.

**Table 1:** Patient characteristics

Patient number	Age at operation	Age at symptom onset	Sex	Etiology	Affected foot
1	49	43	M	IS	R
2	64	61	M	IS	R
3	52	52	M	IS	L
4	52	40	F	MS	L
5	47	44	F	IS	L
6	68	58	F	IS	L
7	50	44	M	ICH	R
8	59	23	F	IS	L
9	57	47	M	MS	R
10	32	27	M	ICH	L
11	42	34	M	ICH	R
12	38	35	F	ICH	R
13	71	68	M	IS	R
14	27	24	F	ICH	R
15	57	56	F	IS	R
16	40	32	F	IS	R
17	76	75	M	IS	L
18	56	53	F	ICH	R
19	64	61	F	ICH	R
20	45	43	M	MS	R
21	53	34	F	IS	R
22	66	62	M	IS	L
23	57	46	M	ICH	R
24	54	52	F	IS	L
25	58	50	F	IS	L
26	59	50	F	IS	L
27	57	48	M	MS	L
28	46	42	F	IS	L
29	38	36	F	ICH	R
30	50	44	M	IS	R
31	25	12	F	ICH	L
32	18	11	F	ICH	R
33	47	45	M	IS	L
34	56	55	F	IS	R
35	42	38	F	ICH	L
36	55	52	M	ICH	R
37	53	51	M	IS	L
38	70	67	M	IS	L
39	53	52	M	IS	R
40	66	63	M	IS	R
41	53	52	M	IS	R
42	55	52	F	ICH	R
43	59	50	M	IS	R
44	58	55	M	IS	R
45	48	39	M	ICH	L

*M* male, *F* female, *ICH* intracerebral hemorrhage, *IS* ischemic stroke, *MS* multiple sclerosis, *L* left, *R* right

The inclusion criteria for the study were the same as the indication criteria for siFES, because this was a retrospective study. Data from all patients who received siFES over the study period were included in the present analyses. The exclusion criteria for the study were the same as the contraindications for siFES for the same reason. These criteria are provided in Table 2. Patients were required to be able to walk at least 20 m with or without a walking aid but without assistance from another person. All patients were tested with tcFES. Eleven patients already used tcFES prior to the initial visit to our clinic. For the remaining 34 of the 45 patients, a pre-operative testing phase was added to establish whether these patients would be likely to profit from peroneal nerve stimulation. Transcutaneous systems applied included MyGait®, Otto Bock, Duderstadt, Germany; NESS®L300, Bioness, Valencia, USA), and Microstim®, Kraut & Timmermann, Hamburg, Germany). Improvement of the gait pattern following tcFES, as rated subjectively by the treating physicians and the patient, resulted in offering surgical intervention to the patient. Benefit from tcFES was assumed when gait speed and distance increased, a reduction in effort while walking was reported, or there was a reduction in falls. If the level of spasticity or degree of contractures prevented adequate range of movement to enable an independent walk of 20 m, siFES was not deemed to be a suitable treatment option.

**Table 2:** Criteria for suitability of siFES to treat drop foot in individual patients. *BMRC* British Medical Research Council. *MS* multiple sclerosis

Indication criteria	Contraindication for siFES
- Benefit from tcFES	- Contracture of ankle
- Drop foot (dorsiflexion or eversion BMRC 0/5–4/5 impairing ambulatory ability)	- High risk for anesthesia
- Range of ankle movement at least 30°	- Pregnancy
- First motor neuron lesion (ischemic or hemorrhagic stroke or MS)	- Uncontrollable epilepsy with grand mal seizures
- Initial event of drop foot at least 6 months previously	- Inability to give informed consent
- Ability to walk 20 m unassisted	- Drop foot due to second motor neuron lesion
- Completed rehabilitation	

Motor function improvement during the 6 months prior to surgery was an exclusion criterion, in order to avoid monitoring of spontaneous recovery. Peripheral neuropathy was excluded by nerve conduction velocity (NCV) (> 38.1 ms).

### Implanted device

The Actigait system® for administration of siFES was employed, because it offers both conceptual and practical advantages over tcFES. Stimulation is mediated via a 4-channel cuff electrode, which is positioned around the peroneal nerve just below the seperation of the peroneal sensory branch and above the kneefold. Each of these channels can be activated separately. The intensity of the activation is controlled by the length of the stimulation impulse, applying the rheobase concept [9, 15, 31]. This approach enables activation of specific fascicles, opening up possibilities for individually-tuned stimulation to provide a potentially more balanced foot lift. Sensory side-effects may potentially be reduced compared to tcFES, where activation of skin fibers cannot be avoided. The externally-worn heel-switch supplies a signal to the steering unit when the heel is lifted from the floor, thus triggering the signal for the internally-worn antenna, initiating activation of the electrode [2].

### Surgery

All surgical procedures were performed under general anesthesia. Patients received a 4 mm diameter 4-channel cuff electrode (Actigait®, Otto Bock, Duderstadt, Germany), which was placed around the peroneal nerve above the knee fold and below the cutaneous branch of the peroneal nerve [2]. After implantation, the leg with the implanted device was partially immobilized using a flexible cast allowing for 30 degree flexion. Platelet inhibitors and coumarine derivatives were stopped for 7 days prior to surgery. Coumarine-treated patients received weight-adapted low molecular weight heparin bridging therapy until the evening before surgery. Post-operative anticoaglulation was initiated individually according to risk for post-operative haemorrhage.

### Activation procedure

Initial activation of the stimulation system was performed after time to allow for complete healing, including development of scar tissue around the electrode, to prevent potential dislocation. A minimum washout phase of at least 4 weeks after surface stimulation was stipulated before determining baseline parameters. Time to activation ranged from 16 to 88 days (mean: 33 days), excluding one patient, in whom the system was activated 790 days post-surgery, after recovery from partial nerve damage. During the activation procedure, each channel of the electrode was initially tested separately, with the patient supine, to define the range of stimulation intensity (width of a single pulse) between the motor threshold and the maximum motor response, as well as the preliminary optimal intensity. Channels mainly leading to pain or to plantarflexion were left deactivated. In the second phase, the remaining channels were tested individually, and in combination under walking conditions, to identify the optimal stimulation intensity and a balanced dorsiflexion, and to define the 7 levels within which the patient would be able to modify stimulation intensity independently while walking. Sensory side-effects were minimized through adjustment of stimulus duration, ranging between 30 and 250 μs, and frequency, from 15 to 45 Hz, at each of the four independent channels,

### Gait speed

A standardized 10 m walking test was performed before and on initiation of continuous FES (T1), and with stimulation on and off after 3 months (T2) and after 1 year (T3). At each evaluation, gait speed was assessed first in the FES OFF and subsequently in the FES ON condition. Mean values over three walking test repetitions were derived for each condition. Measurements were taken with a stopwatch beginning with the third step after initiation of walking. Between measurements, patients rested for 5 min to avoid systematic fatigue effects. There were no breaks between the three repetitions for each condition. Patients were instructed to perform the test first at a comfortable and then at maximum pace.

### SF-36

All patients received the validated SF-36 questionnaire prior to surgery (T1), after 3 months (T2), and after 1 year (T3) of continuous therapy. The SF-36 was designed for evaluating health status in the Medical Outcomes Study [30]. It originally comprised 8 sections, which can be divided into physical functioning (P) and emotional well-being (E). Subsequently, the health change (HC) category was added [11]. The P sections include physical functioning, role limitations due to physical health, bodily pain, and general health. The E sections include emotional well-being, role limitations due to emotional problems, social functioning, and energy/fatigue.

### Assessment of potential side-effects

Sensory side-effects were rated by the patients with a visual analogue scale (VAS), ranging from 0 (no sensation) to 10 (maximal possible pain), initially with tcFES before receiving siFES, and subsequently with siFES at T1, T2, and T3. Local skin irritation was evaluated by physical examination during the follow-up visits at 3 (T2) and 12 (T3) months after system activation. The pre-operative assessment of sensory side-effects was added during the course of the clinical evaluations of these patients and was therefore not available for the first 9 patients. Other complications that arose are also reported.

### Statistics

Two-way repeated measures ANOVAs were performed to evaluate the difference in gait velocity with stimulation ON compared with OFF at the three assessment time points separately for maximum and comfortably-paced gait. Main effects of *stimulation* and *time* were sought, as well as a potential interaction. One-way repeated measures ANOVAs were applied to evaluate changes in total SF-36 score and SF-36 sub-category scores, including a separate evaluation of pain (a part of the physical sub-category of the SF-36), examining potential main effects of *time*. Post hoc pairwise comparisons based on estimated marginal means with Bonferroni correction were applied when a significant main effect of *time* was identified.

The gait velocity, SF-36 total score, and SF-36 physical sub-category data were normally distributed according to the Kolmogorov-Smirnov test for normality (all *p* > 0.05). Examining the effect of time on maximum gait velocity, Maunchly’s test indicated a violation of sphericity, with a Greenhouse-Geisser estimate of sphercity of 0.76. Sphericity was also violated in examining the effect of time on the pain sub-category score, with a Greenhouse-Geisser estimate of sphercity of 0.78. We therefore applied a Huynh-Feldt correction in these cases. All remaining data conformed to the assumption of sphericity.

Pearson’s correlation was calculated between the absolute values of each of the QoL scores evaluated and the absolute maximum and comfortable gait speeds, as well as between the changes in QoL score and changes in speed. Correlation was additionally calculated between the total SF-36 score (with the pain component subtracted) and the pain score.

The sensory side-effects, quantified with the VAS, were compared between prior use of tcFES and using siFES, at each time point, using two-sided paired T-tests.

## Results

Forty-one stroke (15 hemorrhagic, 26 ischemic) and 4 MS patients received surgical implantation of the stimulation system. The age range was 18 to 76 years (mean 52 years). The cohort comprised 24 men and 21 women. The left foot was affected in 19 patients, and in 26 patients, the right foot. The mean time between symptom onset and surgery was 5.9 (std: 6.1) years. Because the study was retrospective, formal screening was not performed. Multiple regional centres were informed of the treatment option of siFES for drop foot in chronic stroke and MS patients and were provided with the pre-requisites and contraindications for this treatment (Table 2). We received referrals of patients who wished to discuss this treatment option, and over the period from 2011 to 2016, 45 patients chose to undergo siFES. We have analysed all available data from all 45 patients. All 45 patients continued to use siFES at 1 year.

For 44 of 45 patients, the system was activated within an interval of 10 days to 4 weeks post-implantation. Maximum gait velocity was recorded with stimulation ON in 43 patients and OFF in 40 patients at T1, ON in 40 and OFF in 39 patients at T2, and both ON and OFF in 39 patients at T3. Comfortable gait velocity was recorded with stimulation ON in 40 and OFF in 39 patients at T1, ON in 39 and OFF in 38 patients at T2, and both ON and OFF in 38 patients at T3. The patients who could not be tested at T1 with the stimulation OFF all required an AFO to mobilize. All available data were included in the statistical analyses. We note here that the condition and time points of the missing measurements varied across patients, such that complete data, with gait velocity ON and OFF for all three time points, were available for 33 patients at maximum velocity and 32 patients at comfortable gait velocity. SF-36 values were available for all 45 patients at T1 and T2 and for 43 patients at T3.

### Gait speed

#### Maximum pace

**Fig. 1:**
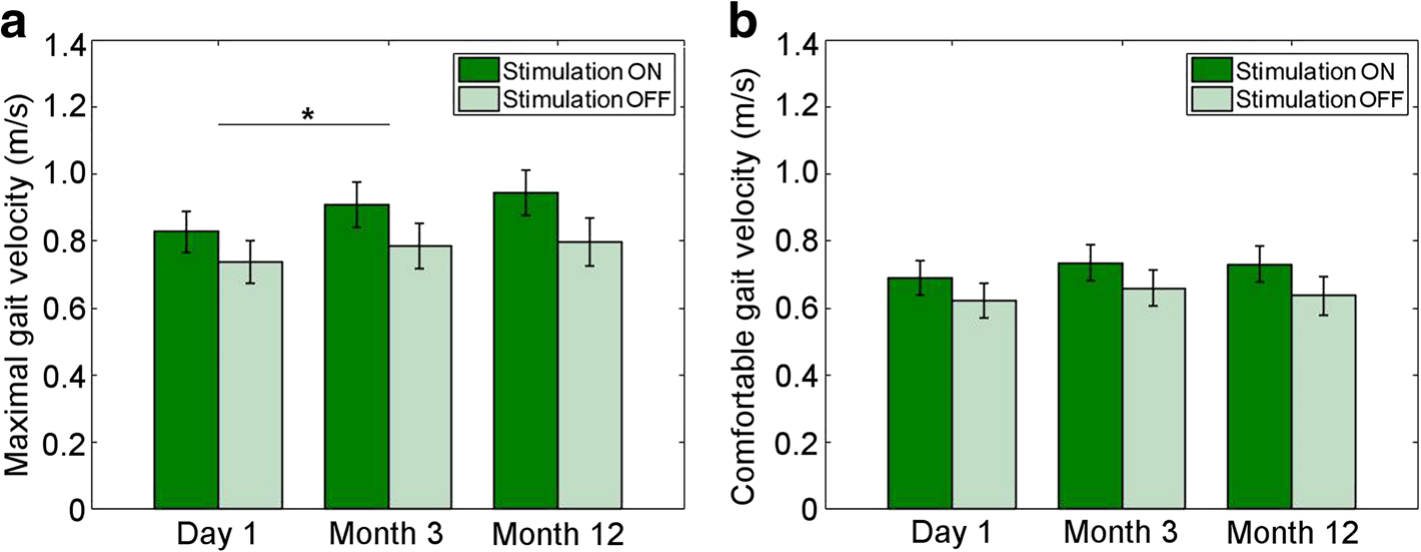
Gait speed (m/s) in relation to duration of therapy with stimulation ON and OFF. **a**. Maximum gait velocity. Main effect of *stimulation* and *time*. Post hoc testing: significant difference from day 1 to month 3 (*). **b**. Comfortable gait velocity. Main effect of *stimulation* only. Error bars = standard error of the mean

A two-way repeated measures ANOVA was applied to the maximum gait velocity, including all participants with measurements both ON and OFF stimulation at all the time points (*N* = 33) (Fig. 1, Table 3). A main effect of *stimulation* was observed (F(1,32) = 58.6, ***p*** **< 0.001,** partial η^2^ = 0.65), with a greater maximum gait velocity across all time points with the stimulation ON (0.92 m/s) than OFF (0.79 m/s). Stimulation ON was thus associated with a mean increase in maximum gait velocity of 0.13 m/s (95% CI: 0.094 to 0.16, p < 0.001). A main effect of *time* was also observed (F(1.6,50.3) = 5.07, ***p*** **= 0.015,** partial η^2^ = 0.14). Post hoc analysis with a Bonferroni adjustment, taking the ON and OFF stimulation conditions together, revealed a significant increase in maximum speed from T1 to T2 of 0.055 m/s (95% CI: 0.013 to 0.097, ***p*** **= 0.012**) and from T1 to T3 of 0.096 m/s (95% CI: 0.022 to 0.17, ***p*** **= 0.012**), with a non-significant increase from T2 to T3 of 0.041 m/s (95% CI: − 0.024 to 0.106, ***p*** **= 0.21**). There was no significant interaction between *stimulation* and *time* (F(2,64) = 1.91, *p* = 0.16, partial η^2^ = 0.056). Excluding the patients with MS did not change which findings were significant.

**Table 3:** Maximum gait speed in m/s during 10 m walking test at all three follow-up evaluations. Mean (std). (*N* = 33)

	1 day post-op (T1)	3 months post-op (T2)	12 months post-op (T3)
Stimulation on	0.84 (0.42)	0.91 (0.44)	0.94 (0.43)
Stimulation off	0.75 (0.42)	0.79 (0.41)	0.82 (0.45)

#### Comfortable pace

A two-way repeated measures ANOVA was also applied to the comfortable gait velocity, including all participants with measurements both ON and OFF stimulation at all the time points (*N* = 32) (Fig. 1, Table 4). When patients were asked to walk at a comfortable pace, a main effect of whether stimulation was ON or OFF was observed (F(1,31) = 40.4, ***p*** **< 0.001,** partial η^2^ = 0.57), with a greater comfortable gait velocity across all time points with the stimulation ON (0.75 m/s) than OFF (0.66 m/s). Stimulation ON was thus associated with a mean increase in maximum gait velocity of 0.083 m/s (95% CI: 0.056 to 0.109, ***p*** **< 0.001**). There was no main effect of *time* (F(2,62) = 2.58, ***p*** **= 0.084,** partial η^2^ = 0.077) and no significant interaction between *stimulation* and *time* (F(2,62) = 0.76, *p* = 0.47, partial η^2^ = 0.024). Excluding the patients with MS did not change which effects were significant.

**Table 4:** Comfortable gait speed in m/s during 10 m walking test at all three follow-up evaluations. Mean (std). (*N* = 32)

	1 day post-op (T1)	3 months post-op (T2)	12 months post-op (T3)
Stimulation on	0.71 (0.34)	0.76 (0.34)	0.76 (0.35)
Stimulation off	0.64 (0.33)	0.68 (0.34)	0.67 (0.37)

#### SF- 36

**Fig. 2:**
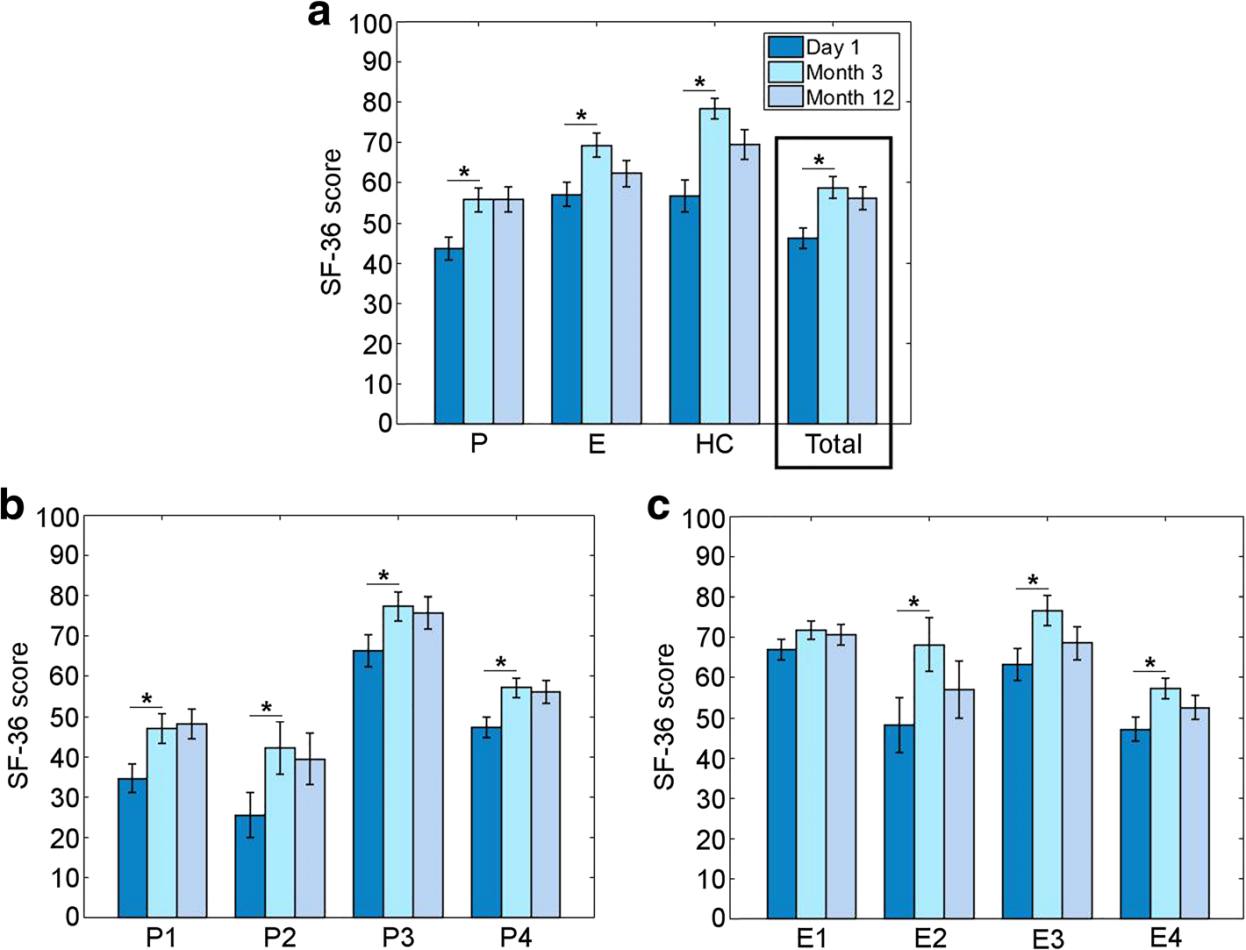
Impact on quality of life over time evaluated using the SF-36 questionnaire. **a**. Mean SF-36 scores. The mean total scores are highlighted with a black box. P = physical health; E = emotional health; HC = health change. **b**. Scores for physical health categories: P1 = physical functioning; P2 = role limitations due to physical health; P3 = pain; P4 = general health. **c**. Scores for emotional health categories: E1 = emotional well-being; E2 = role limitation due to emotional problems; E3 = social functioning; E4 = energy/fatigue. Error bars = standard error of the mean. Main effect of *time* in all sub-categories except E1. * = significant on post hoc testing

Taking the total SF-36 scores at T1, T2, and T3, a main effect of *time* was observed (*N* = 43, F(2,84) = 17.9, ***p*** **< 0.001,** partial η^2^ = 0.30) (Table 5, Fig. 2). The greatest change in total SF-36 score took place between T1 and T2, with a mean of 11.7 (95% CI: 6.70 to 16.70, **p < 0.001**) points more at T2. From T2 to T3, there was no significant change in score. Indeed there was a slight decrease in score, but the difference between T1 and T3 remained significant, with a mean of 9.67 (95% CI: 4.15 to 15.2, ***p*** **< 0.001**) points more at T3.

**Table 5:** Quality of Life according to the SF-36 evaluation

SF-36 Category	Time of evaluation	Mean score (standard deviation)	Number of participants (N)
Physical	T1	43.6 (18.6)	45
	T2	55.8 (20.0)	45
	T3	56.8 (21.0)	43
Pain score	T1	66.3 (27.3)	45
	T2	77.6 (23.5)	45
	T3	76.7 (26.8)	43
Emotional	T1	57.6 (20.1)	45
	T2	69.0 (20.0)	45
	T3	63.0 (22.5)	43
Health change	T1	56.7 (26.3)	45
	T2	78.3 (17.4)	45
	T3	68.6 (25.6)	43
Total score	T1	46.2 (16.8)	45
	T2	58.8 (17.9)	45
	T3	57.0 (19.2)	43

Taking the SF-36-P sub-category scores at T1, T2, and T3, a main effect of *time* was observed (*N* = 43, F(2,84) = 17.2, **p < 0.001,** partial η^2^ = 0.29) (Table 5, Fig. 2). The greatest change in total SF-36 score took place between T1 and T2, with a mean of 11.9 (95% CI: 6.51 to 17.3, **p < 0.001**) points more at T2. The difference between T1 and T3 was also significant, with a mean of 12.2 (95% CI: 6.00 to 18.4, ***p*** **< 0.001**) points more at T3, but the further increase in score from T2 to T3 was not significant.

Taking the SF-36-P-pain sub-category scores at T1, T2, and T3, a main effect of *time* was observed (*N* = 43, F(1.61,67.7) = 4.95, ***p*** **= 0.015,** partial η^2^ = 0.11) (Table 5, Fig. 2). The greatest change in pain score took place between T1 and T2, with a mean of 10.1 (95% CI: 4.38 to 17.6, ***p*** **< 0.001**) points more at T2. Neither the difference between T1 and T3 nor between T2 and T3 was significant.

Taking the SF-36-E sub-category scores at T1, T2, and T3, a main effect of *time* was observed (N = 43, F(2,84) = 5.82, ***p*** **= 0.004,** partial η^2^ = 0.12) (Table 5, Fig. 2). The greatest change in total SF-36 score took place between T1 and T2, with a mean of 10.2 (95% CI: 3.48 to 16.85, ***p*** **= 0.001**) points more at T2. The difference between T1 and T3 and between T2 and T3, were not significant.

Taking the SF-36-HC sub-category scores at T1, T2, and T3, a main effect of *time* was observed (N = 43, F(2,84) = 17.9, ***p*** **< 0.001,** partial η^2^ = 0.30) (Table 5, Fig. 2). The greatest change in total SF-36 score took place between T1 and T2, with a mean of 11.7 (95% CI: 6.70 to 16.70, ***p*** **< 0.001**) points more at T2. The difference between T1 and T3 was also significant, although the improvement was smaller, with a mean of 9.67 (95% CI: 4.15 to 15.2, ***p*** **< 0.001**) points more at T3. Indeed, from T2 to T3, there was no significant change in score.

### Correlations

The total SF-36 test score correlated with maximum gait speed at T2 with the stimulation ON (*N* = 40, Pearson’s r = 0.49, ***p*** **= 0.001**) and also OFF (*N* = 39, r = 0.51, ***p*** **= 0.001**) and also at T3 (N = 39, ON: r = 0.38, ***p*** **= 0.020**; OFF: N = 39, r = 0.36, ***p*** **= 0.026**) (Table 6). Analogous correlations were seen with comfortable gait speed at T2 (ON: N = 39, r = 0.47, ***p*** **= 0.002**; OFF: *N* = 38, r = 0.48, ***p*** **= 0.002**) and T3 (ON: N = 38, r = 0.38, ***p*** **= 0.020**; OFF: N = 38, r = 0.35, ***p*** **= 0.029**) (Table 7).

**Table 6:** Correlations between SF-36 total score and sub-scores and maximum gait speed. Excluding the MS patients changed the following: Total SF-36 and T1 with stimulation OFF: *r* = 0.29, *p* = 0.083, *N* = 37. SF-36-P and T1 with stimulation OFF: *r* = 0.28, *p* = 0.093, *N* = 37. SF-36-E and T2 with stimulation ON: *r* = 0.30, *p* = 0.073, *N* = 37. SF-36-E and T1 with stimulation OFF: *r* = 0.25, *p* = 0.13, *N* = 37; SF-36-E. * = p < 0.05

QoL	T1 (Stimulation OFF)	T2 (Stimulation ON)	T3 (Stimulation ON)
Total SF-36	*r* = 0.37	*r* = 0.50	*r* = 0.38
	*p* = 0.018*	*p* = 0.001*	*p* = 0.020*
	*N* = 40	*N* = 40	*N* = 39
SF-36-P	*r* = 0.36	*r* = 0.39	*r* = 0.21
	*p* = 0.022*	*p* = 0.013*	*p* = 0.19
	*N* = 40	*N* = 40	*N* = 39
Pain score	*r* = 0.12	*r* = − 0.18	*r* = − 0.11
	*p* = 0.44	*p* = 0.27	*p* = 0.49
	*N* = 40	*N* = 40	*N* = 39
SF-36-E	*r* = 0.33	*r* = 0.32	*r* = 0.37
	*p* = 0.038*	*N* = 40	*p* = 0.023*
	*N* = 40	*p* = 0.043*	*N* = 39
Health change	*r* = − 0.028	*r* = 0.23	*r* = − 0.055
	*p* = 0.87	*p* = 0.15	*p* = 0.74
	*N* = 40	*N* = 40	*N* = 39

**Table 7:** Correlations between SF-36 total score and sub-scores and comfortable gait speed. Excluding the patients with MS (reducing to *N* = 36 or *N* = 35) did not alter which correlations were significant. * = *p* < 0.05

QoL	T1 (Stimulation OFF)	T2 (Stimulation ON)	T3 (Stimulation ON)
Total SF-36	*r* = 0.26	*r* = 0.47	*r* = 0.38
	*p* = 0.11	*p* = 0.002*	*p* = 0.020*
	*N* = 39	*N* = 39	*N* = 38
SF-36-P	*r* = 0.20	*r* = 0.41	*r* = 0.23
	*p* = 0.23	*p* = 0.009*	*p* = 0.17
	*N* = 39	*N* = 39	*N* = 38
Pain score	*r* = − 0.005	*r* = − 0.17	*r* = − 0.13
	*p* = 0.98	*p* = 0.29	*p* = 0.44
	*N* = 39	*N* = 39	*N* = 38
SF-36-E	*r* = 0.26	*r* = 0.25	*r* = 0.36
	*p* = 0.12	*p* = 0.12	*p* = 0.028*
	*N* = 39	*N* = 39	*N* = 38
Health change	*r* = − 0.037	*r* = 0.20	*r* = − 0.079
	*p* = 0.83	*p* = 0.24	*p* = 0.64
	*N* = 39	*N* = 39	*N* = 38

**Table 8:** Pearson’s correlation between change in gait velocity at maximum and comfortable pace and change in SF-36 score and its sub-categories over the corresponding time period with the stimulation ON. All patients for whom the ANOVAs were calculated were included. Note that the findings were unaltered when the two patients with MS with complete data available were excluded. * = *p* < 0.05

	Maximum gait velocity			Comfortable gait velocity		
	T1 to T2	T2 to T3	T1 to T3	T1 to T2	T2 to T3	T1 to T3
Total SF-36	*r* = 0.11	*r* = 0.16	*r* = 0.057	*r* = 0.22	*r* = 0.17	*r* = 0.36
	*p* = 0.53	*p* = 0.38	*p* = 0.75	*p* = 0.23	*p* = 0.35	*p* = 0.045*
SF-36-P	*r* = 0.028	*r* = 0.15	*r* = 0.091	*r* = 0.21	*r* = 0.095	*r* = 0.38
	*p* = 0.88	*p* = 0.41	*p* = 0.62	*p* = 0.25	*p* = 0.61	*p* = 0.033*
Pain score	*r* = − 0.19	*r* = 0.22	*r* = 0.17	*r* = − 0.13	*r* = − 0.17	*r* = 0.078
	*p* = 0.28	*p* = 0.22	*p* = 0.33	*p* = 0.48	*p* = 0.36	*p* = 0.67
SF-36-E	*r* = − 0.030	*r* = 0.029	*r* = 0.090	*r* = − 0.076	*r* = − 0.032	*r* = 0.21
	*p* = 0.87	*p* = 0.87	*p* = 0.62	*p* = 0.68	*p* = 0.86	*p* = 0.26
Health change	*r* = 0.020	*r* = 0.11	*r* = 0.19	*r* = 0.030	*r* = 0.30	*r* = 0.13
	*p* = 0.903	*p* = 0.50	*p* = 0.28	*p* = 0.86	*p* = 0.076	*p* = 0.46

The SF-36 sub-category scores for physical and emotional QoL both correlated with maximum gait velocity at T1 and T2, but only the emotional score correlated at T3. The emotional QoL score also correlated with comfortable gait velocity at T3.

The increase in comfortable gait velocity from T1 to T3, including all available participants (*N* = 32), also correlated both with the improvement in total SF-36 score (r = 0.36, ***p*** **= 0.045**), as well as with improvement in the physical subscore of the SF-36 (r = 0.38, ***p*** **= 0.033**) (Table 8). These findings were unaltered when the patients with MS were excluded (*N* = 30, r = 0.36, ***p*** **= 0.049**; r = 0.38, ***p*** **= 0.037**). The increase in maximum gait velocity, however, did not correlate with the improvement in QoL score, including total SF-36, as well as the P, E, and pain sub-categories separately, whether or not the patients with MS were included (*N* = 33; *N* = 31).

The pain score did not correlate with the maximum or the comfortable gait velocity with stimulation ON or OFF at any of the three time points, and there was also no correlation between change in pain score and change in maximum or comfortable gait velocity. On the other hand, the pain score did correlate with the overall SF-36 score (from which the pain score was first subtracted), at T1 (*N* = 41, r = 0.79, ***p*** **< 0.001**) and at T2 (N = 41, r = 0.63, **p < 0.001**) but not at T3.

### Sensory side-effects and skin lesions

All patients used a surface stimulation system in a testing session prior to surgery. Sensory side-effects were evaluated in 27 of the patients using tcFES and in 36 patients using siFES, with ratings available at T1 from 29 patients, at T2 from 30 patients, and at T3 from 28 patients. Compared to using tcFES (mean: 5.7, std 1.9), side-effects were rated as 56% lower using siFES at T1 (mean: 2.5, std: 1.7, T = 7.0, ***p*** **< 0.0001**, *N* = 24), 60% lower at T2 (mean: 2.3, std: 1.9, T = 6.7, ***p*** **< 0.0001**, *N* = 23), and 70% lower at T3 (mean: 1.7, std: 1.7, T = 7.9, ***p*** **< 0.0001**, *N* = 19). Note that the differing patient numbers is due to missing values being scattered across patients. In two patients, a pre-operative testing phase could not be performed, despite good motor response, due to substantial pain when applying tcFES. None of the patients with implanted electrodes perceived the remaining stimulus-induced paresthesia as debilitating.

Using surface electrodes (tcFES), 11 patients experienced skin lesions, ranging from painful erythema to superficial burns. With siFES, one patient developed a mild transient erythema beneath the fixation patch.

### Adverse events

One patient required revision surgery due to a pronounced subcutaneous hematoma resulting from strict anticoagulant management. A further patient underwent revision after 15 months due to clinical suspicion of cuff loosening. 3 months later, the patient showed improved foot lift, with adequately balanced dorsiflexion.

Another patient showed signs of a mild sensory axonal neuropathy pre-operatively, with reduced impulse amplitude in the sural nerve but intact peroneal NCV. He developed axonal nerve damage, possibly due to postoperative swelling of the peroneal nerve. The patient did not undergo revision, as electrophysiological examination demonstrated axonal damage. Wallerian degeneration and nerve regrowth were anticipated. After 24 months, the system was switched on. Long-standing lymphedema of the paretic leg was markedly reduced after 3 months.

## Discussion

The use of siFES led to increased gait velocity and improved QoL in a large cohort of predominantly chronic stroke and also MS patients. Most of the improvements in both gait speed and QoL occurred over the first 3 months following commencement of stimulation. At long-term 12-month follow-up, these improvements were maintained, but no further improvement was observed over this longer time period suggesting that 3 months provided an adequate follow-up period for evaluating the impact of stimulation on gait speed. In accordance with our hypothesis, increases in gait speed correlated with improvements in QoL. However, the findings differed between maximum and comfortable gait speed and between the QoL evaluation physical and emotional sub-categoies. Correlation between gait velocity and QoL is rarely addressed in the FES literature, but we observed significant correlations between these parameters at 3- and 12-month follow-up, both in absolute terms and in terms of changes in these parameters over time.

While maximum gait velocity significantly increased over time, the increase in comfortable gait velocity over time was not significant. These observations suggest that the stimulation enabled participants to walk at their personal target speed, and although it supported further speed increases, these higher speeds were not sought for normal walking.

The improvement in QoL over the study period, which was significant from day 1 to month 3 and maintained at 12 months but without further improvement, was also seen in the physical and health change sub-categories separately. This pattern was moreover conserved at the level of further sub-categorization within these sub-categories, with an improvement from T1 to T2, which was maintained but not further increased by T3. The exception was the emotional sub-category, in which the score improved from T1 to T2 but no longer from T1 to T3, with an insignificant reduction from T2 to T3. The improvement in emotional aspects of QoL was therefore observed over the first 3 months, which is the time period over which gait speed also most improved. E1, which measures emotional well-being, remained stable throughout the study period. In contrast, E2, which reflects role limitation due to emotional problems, improved from T1 to T2. The difference may be interpreted as resulting from an increase in autonomy. As predicted, the pain sub-score improved with therapy, but while the score improved from T1 to T2, by T3, there was no significant difference to reports at T1.

A correlation between pain and non-physiological joint parameters has been described for MS patients [12]. We speculate that the reduction in pain in the current study over the first 3 months (T1 to T2) could be explained by a more balanced gait pattern [10], with avoidance of overexertion of the unaffected half of the body. Pain may have been again reported at T3, with no further change in their conditions from T2 to T3, as it had only improved at T2 rather than been eliminated. Another possibility is that some pain returned after 12 months (by T3) due to increased walking distance or higher walking speed.

While the pain score correlated with the overall QoL score at T1 and T2, surprisingly, the significant reduction in reported pain seen from baseline assessment to T2 was independent of absolute gait velocity and gait velocity increase across the patient cohort. The correlation of pain score (which did significantly improve with siFES) with the overall QoL score (which also improved significantly with FES), suggests that siFES has a positive effect in eliminating pain, resulting in improvement in QoL that is not necessarily reflected in a faster gait. This finding suggests that evaluation of pain provides an independent outcome parameter affecting QoL and would be a valuable addition to standard outcome measures.

Absolute total QoL scores correlated with absolute maximum and comfortable gait speed after 3 and 12 months with stimulation on, suggesting that the ability to locomote at particular speeds has an impact on QoL. The total QoL score correlated with the maximum gait velocity at all three time points and with the comfortable gait velocity at 3 and 12 months. These findings provide an indirect indication that a faster gait speed is associated with enhanced QoL. Examining the SF-36 sub-categoies, at T1 and T2, maximum gait velocity correlated with both the physical and emotional, and at T3, only the emotional sub-category, while comfortable gait velocity correlated with the physical sub-category at T2 and the emotional at T3. Over the shorter term, the gait speed tended to be more important in physical aspects of QoL, while over the longer term, gait speed was more important in emotional aspects of QoL. Initially, maximum gait speed was more closely associated with QoL, but over time, comfortable gait speed also had an impact on QoL.

In order to evaluate potential direct associations between stimulation-related increases in gait speed and improvement in QoL, we examined parallel changes in QoL and gait speed parameters. While the improvement in both the total QoL score and the physical subscore correlated with the increase in comfortable gait speed over the 1 year follow-up period, the increase in maximum gait velocity did not correlate with the degree of QoL improvement. That is, although a greater QoL is associated with a higher maximum gait speed, a greater improvement per se in the maximum gait speed does not necessarily result in a comparable QoL enhancement. These findings suggest that comfortable gait velocity is a potentially more important determinant of QoL than the maximum gait speed. Together with the absence of a continued increase in comfortable gait speed, we tentatively suggest that participants tended to reach their target comfortable gait speed at first use of siFES and that this led to improved QoL.

Our findings support our hypothesis that there is a relationship between gait speed and QoL. Gait speed is deemed to provide an adequate surrogate marker for evaluating therapeutic effects of FES in central drop foot, because it correlates with the ability to cover meaningful distances in the community [21, 24]. Gait velocity has also been found to be inversely correlated with the risk of falling, with the suggestion that slow walkers experience more falls and more fear of falling [8]. A minimum change of 0.05 m/s in gait velocity has been determined to be clinically meaningful and 0.1 m/s as substantial [20]. We observed a mean increase of 0.23 m/s in maximum and of 0.12 m/s in comfortable gait velocity from the initial OFF to the final ON condition. Moreover, the maximum and comfortable gait velocities with the stimulation ON after 3 months and also after 12 months were found to correlate with the total score on the QoL assessment across individuals in the current cohort.

A non-significant further increase in velocity after 12 months compared to 3 months means that an improvement partly resulting from training cannot be excluded, including a potential motor learning component. While a carry-over effect was previously noted in an MS patient using implantable FES for over 1 year, enabling the patient to be mobile in his home in the OFF condition without using an orthotic device [10], no therapeutic effect of implantable FES was observed in a cohort of chronic stroke patients after 6 months [14]. In a meta-analysis of four tcFES studies, Robbins et al. [22] described a therapeutic effect in stroke patients in the chronic phase. The differing findings might be explained by the considerable difference with respect to the timing of intervention after stroke (109 months vs. 37 months) between the study by Kottink et al. and the meta-analysis. In the current study, the surgery took place at a mean of 71 months after stroke, and ongoing spontaneous recovery was ruled out by exclusively including patients with stable motor deficit during the preceding 6 months. Furthermore, our findings render the 3 month outcome a clinically meaningful study endpoint, which is potentially relevant for the design of future trials.

Recent evidence suggests that the prolonged use of implantable peroneal FES could enhance cortical plasticity in some patients, reflected by shifts of activated fields in the sensory cortex, as measured using MEG [18], and by normalisation of activity in the pre-motor area detected using FDG-PET [29]. Both phenomena could be interpreted as cortical relearning. The size of the MEG effect correlated with cadence, a relevant parameter for gait analysis [18]. It was argued that patients who maintain gait improvments for a longer time after the device is turned off may not need further therapy. In the current cohort, which was partially included in the series described by Merkel et al. [18], there was no interaction between stimulation and time. In this larger cohort, even after long-term FES, cortical relearning and muscular training effects alone were not sufficient to increase gait velocity significantly in the absence of continued stimulation on a group level.

TcFES has been shown to increase gait speed both in patients post-stroke [22] and in patients with MS [5, 19]. While tcFES is a valid treatment option for many drop foot patients (see [4] for a review), handling problems, difficulties in finding optimal electrode positions, and skin lesions associated with surface stimulation can become obstacles for therapy adherence. Indeed, many of our patients reported some difficulties in handling surface tcFES, due to concomitant upper limb paresis. These handling problems were diminished once therapy with siFES was initiated. We identified two patients in this cohort who were unable to tolerate surface FES for more than a walking test. Both tolerated siFES well over the year following implantation.

Taylor et al. examined the influence of a different siFES system, describing a significant increase in walking speed, walking distance, and maintenance of walking speed gain after 3 years in 23 MS patients [28], consistent with the present findings. In addition, they reported higher therapy adherence when comparing the implantable device to external FES, which we did not explicitly investigate in our series. We note, however, that the continued use of siFES in all 45 of our patients at the one-year follow assessment shows high therapy adherence.

### Limitations

Limitations include the retrospective study design and the clinically mixed population. We note, however, that the significant effects of stimulation and time on gait velociy were not altered when the data from the patients with MS were excluded from the analysis. Indeed, in a study directly comparing the effects of tcFES on walking speed between patients with non-progressive (e.g., post-stroke) and progressive (e.g., patients with MS) disorders, the increase in speed after 3 months did not differ between the groups [25]. Although follow-up at 11 months showed no significant difference between the groups, a slight further increase in speed was seen in the non-progressive group and a plateau in the progressive group. The authors concluded that the foot drop itself was more important than the diagnosis and pointed out that disease progression itself may account for the slightly different courses over the longer term. We note, moreover, that at 5 year follow-up, patients with MS continued to have an orthotic effect with tcFES [26].

A further limitation is that we recorded a limited number of outcome measures. Potential improvement in motor control (strength, motor selectivity), sensibility, range of ankle movement, and change in spasticity scores over time were not evaluated. Future work should moreover include walking distance, gait pattern and efficiency, and formal recording of patient experiences, as well as direct comparison with the use of an AFO in the same patient group.

We also note that certain data were missing. One patient was unable to walk without walking aids at T1, and another patient at both T1 and T2, so that the OFF measurements could not be performed. Because of the risk of falling, the maximum velocity gait test was not performed in an additional patient. Other data were missing due to lack of measurement of comfortable gait velocity, because that condition was added later to the protocol.

For patient recruitment, we educated doctors and physiotherapists in regional rehabilitation institiutions regarding the possibility of providing an implanted stimulation system to improve central drop foot. This approach might have resulted in a selection bias towards patients who were a priori poor candidates for tcFES or orthotic devices. All patients seen in our institution had been equipped with an orthotic device and were not satisfied with the results. However, we did not compare orthotic devices and FES directly. Moreover, we did not ask whether patients were more satisfied using tcFES than their orthotic device.

## Conclusions

In summary, siFES is an effective and safe treatment option for central foot drop and can lead to high adherence to therapy. The present results clearly show that peroneal FES using implantable electrodes leads to increased gait speed and can provide meaningful improvements in the quality of life of patients with central foot drop.

## Data Availability

All data generated or analyzed during this study are included in this published article.

## Abbreviations

FES: Functional Electrical Stimulation
MS: Multiple Sclerosis
NCV: Nerve conduction velocity
QoL: Quality of Life
SF-36: Short form 36
siFES: Semi-implantable Functional Electrical Stimulation
std: Standard deviation
tcFES: Transcutaneous Functional Electrical Stimulation
